# Modeling the Internet of Things, Self-Organizing and Other Complex Adaptive Communication Networks: A Cognitive Agent-Based Computing Approach

**DOI:** 10.1371/journal.pone.0146760

**Published:** 2016-01-26

**Authors:** Samreen Laghari, Muaz A. Niazi

**Affiliations:** 1 Computer Science Department, Islamabad Model College for Girls, Street #25, F-6/2, Islamabad, Pakistan; 2 Computer Science Department, COMSATS Institute of IT, Park Road, Islamabad, Pakistan; University of Maribor, SLOVENIA

## Abstract

**Background:**

Computer Networks have a tendency to grow at an unprecedented scale. Modern networks involve not only computers but also a wide variety of other interconnected devices ranging from mobile phones to other household items fitted with sensors. This vision of the "Internet of Things" (IoT) implies an inherent difficulty in modeling problems.

**Purpose:**

It is practically impossible to implement and test all scenarios for large-scale and complex adaptive communication networks as part of Complex Adaptive Communication Networks and Environments (CACOONS). The goal of this study is to explore the use of Agent-based Modeling as part of the Cognitive Agent-based Computing (CABC) framework to model a Complex communication network problem.

**Method:**

We use Exploratory Agent-based Modeling (EABM), as part of the CABC framework, to develop an autonomous multi-agent architecture for managing carbon footprint in a corporate network. To evaluate the application of complexity in practical scenarios, we have also introduced a company-defined computer usage policy.

**Results:**

The conducted experiments demonstrated two important results: Primarily CABC-based modeling approach such as using Agent-based Modeling can be an effective approach to modeling complex problems in the domain of IoT. Secondly, the specific problem of managing the Carbon footprint can be solved using a multiagent system approach.

## Introduction

Modern technological supply chains offer a rapid channel for consumer technology creation and adoption. As new types of technology enters the everyday lives globally, these newer concepts offer new challenges [1]. Practical examples of concepts becoming reality include "PhoneBloks" a phone based on highly modular connected components [2], the Internet of Things (IoT) [3] and cloud computing [4, 5]. These technologies have all become not just commonplace in the Information Technology industry but also in various fields of scientific research. Researchers from fields as diverse as Bioinformatics [6] and astronomy [7] have started to use technology to solve scientific problems in a better way.

As technology becomes commonplace, unfortunately, the human race is expanding its use without completely realizing its implications. We live in a consumer-centric society where manufacturers focus on building devices which sell. The actual implications of the technology, its impact on the environment, the planet and its inhabitants seems to be the least of the concerns. The key problem here is that to be able to understand complex systems, we need to be able to model and simulate them [8].

Electronics in general, and personal computers, in particular, are well-known for their long-term detrimental effects on the environment [9]. Not only do computers significantly add to an ever-increasing pile of waste, yearly usage of a single desktop personal computer can produce around *220* kilos of carbon dioxide (CO_2_) [10]. Industrial whitepapers, such as by the International Data Corporation (IDC) [11] have noted that the environmental damage due to personal computers is of the same order as the environmental impact of the entire airline industry. Likewise, the Australian Computer Society (ACS) [12] has reported that the use of ICT by Australian businesses generates close to 8 million tons (Mt) of carbon emissions yearly. Besides the environmental impact of 20 million tons of carbon emissions, it has been estimated that US businesses spend an extra 2.8 billion USD for keeping computers operational (Including power usage and air conditioning) even in off hours due to reasons such as for the installation of overnight patches [13].

A deceptively simple solution for solving this management problem of power utilization has been proposed by Munzi [14]. Munzi analyzes three projects and notes that simply turning computers off can actually be one of the most effective policies for saving power in the corporate world. Likewise, a similar solution has been proposed by Gartner [15] and others such as the US Department of Energy [16].

While turning computers off could be an effective strategy, unfortunately it is impractical in the corporate world. Any new policy should not cause more problems for an already struggling corporation in the recession-hit global economy. While, in theory at least, it may be easy to control and cut the use of some residential computers by this means, it is not enforceable globally. This is especially true in active corporations with various segments of the workforce in crunch at all times.

### Problem Statement

In previous work, we have proposed the use of modeling and simulation to reduce power usage and to model corporate footprint [17, 18]. However, to the best of our knowledge, previous work has not been performed on modeling the Carbon footprint usage for networks of an entire corporation using Cognitive Agent-based Computing approach [19].

### Research Contributions

In this paper, we model a self-adaptive architecture for managing the Carbon footprint in a corporate environment using a heterogeneous multiagent system. Our key contribution is to model this system using Cognitive Agent-based Computing (CABC). We also illustrate how the Exploratory Agent-based Modeling (EABM) level of the CABC framework helps in modeling complex systems. This modeling exercise demonstrates the modeling of an autonomous system. The proposed system monitors and controls carbon footprint use in a complex network. The system architecture of the proposed Multiagent System for Managing Carbon Footprint (MASMINC) is also presented. MASMINC can be implemented by using a set of cooperative software agents in any corporate network. We show how MASMINC can be useful in management and minimization of corporate Carbon footprint.

### Research Scope and Validation Details

To summarize, the key goal of the paper is to present the modeling exercise for MASMINC using EABM of the CABC framework. MASMINC agents work to reduce the carbon footprint utilization by a mix of following measures:

1. Acquire corporate rules policy from a centralized server.
2. Report individual computer usage to a central server (Allowing for a real-time analysis of the network measured in terms of depletion of allocated Carbon footprint)
3. Identify idle computers on the network by monitoring human interactions.
4. Perform analysis of all computers by making inferences based on rules in light of the usage by the computer user.
5. Decide between different types of computer shutdown policies such as stand-by, hibernation and power off, using assigned policies by the network administrator or the local computer user.
6. In case of a complete or partial exhaustion of assigned corporate footprint for a given network, agents can also be appropriately engineered to notify the network administrator to make further contingency decisions based on the usage.

For an effective validation of the architecture, we develop an agent-based simulation model based on typical personal computer usage platforms in corporate environments (Such as lunch times, schedule of people arriving and leaving offices etc.). The simulation is conducted for networks up to *1000* computers for a number of different scenarios. In all cases, the results demonstrate the effectiveness and scalability of the MASMINC approach in reducing carbon footprint usage–as compared with existing approaches such as the Energy-Star.

### Outline

The rest of the paper is structured as follows:

In the next section, we present necessary background and introduce terms as well as discuss relevant literature. Next, we present the architecture of the proposed multiagent system and the design of the Exploratory agent-based model used for validation of the architecture. This is followed by a detailed discussion of results including suggestions for a practical implementation of the work. Finally, we conclude the paper giving proposals for future directions.

## Background

In this section, we present background for developing a better understanding of the proposed architecture.

### Carbon Footprint

The notion of a carbon footprint is a commonly comprehensible term in relation to the role of emission of Carbon-related gases in the environment. Wiedmann et al. [20] notes that while the term itself is in common usage starting from the concept of ecological footprint by Wackernagel and Rees [21], there is considerable confusion regarding its exact nature. Keeping this in mind, they have introduced a systematic definition of “carbon footprint” to sort out practical questions such as system boundaries, completeness, comprehensiveness, units and robustness of the indicator given as follows:

Definition: “The carbon footprint is a measure of the exclusive total amount of carbon dioxide emissions that is directly and indirectly caused by an activity or is accumulated over the life stages of a product.”

Weber et al. [22] have investigated the global and distributional facets of American household carbon footprint using consumer expenditure surveys and multi-country life cycle assessment techniques. It is pertinent to mention here that Murugesan [23] has noted that a single personal computer can produce a ton of Carbon dioxide yearly.

### Agent-based Computing

Agent-based computing involves the use of agents in different computing scenarios. The use of Agent-based computing ranges from (software) multiagent systems, which are software systems consisting of several agents in a network, to agent-based models [24, 25]. Furthermore, as part of a recent Special Issue of the journal Simulation, Niazi and Hussain have collected papers on the use of agent-based computing in Complex Adaptive COmmunicatiOn Networks and environmentS (CACOONS) [26, 27]. These papers demonstrate the utility of agent-based models to model various systems ranging from distributed computing systems to multiagent systems and sensor networks [28]. Agents in a multiagent system are often classified according to various criteria and attributes such as being intelligent, autonomous, mobile [29], reactive or rational [30].

### Cognitive Agent-based Computing

Cognitive Agent-based Computing Framework was presented by Niazi and Hussain in [19]. The idea is to be able to take any real-world complex system and convert it into a suitable model by choosing a particular modeling level [31]. A particularly notable use of Agent-based Modeling (ABM) and the CABC framework is to allow for the validation of large-scale multiagent systems, which would perhaps otherwise have been impossible to validate before construction [32]. This particular problem arises because during building software agents, it is difficult to foresee the exact nature of interactions of agents as the number of agents grows considerably large. Several case study examples of use of CABC have been presented in literature such as in [31, 33–35].

ABM has been formally discussed in detail by Miller and Page in [36] in the domain of Complex Adaptive Systems (CAS). Furthermore, North and Macal present ABM in the context of businesses [37]. Likewise, Railsback and Grimm, have presented a practical approach to ABM using NetLogo in [38]. Gilbert and Troitzsch present the use of NetLogo and other tools for developing ABM for social simulation [39]. Modeling and simulation is an important part of modern science tools because it allows for prediction and explanation of phenomena which would otherwise not have been possible. Recent examples of such uses include work by Kiparsky et al. [40].

## Model Development

This section first presents the proposed multiagent architecture including various possible mechanisms for the allocation of the distributed Carbon Footprint Allowance (CFPA). Here, it is pertinent to note that the term CFPA has been used to refer to the total usable amount of carbon footprint allocated to a given network. This is followed by the design of the Exploratory agent-based model design using CABC framework.

### Architecture Goal

**Fig 1:**
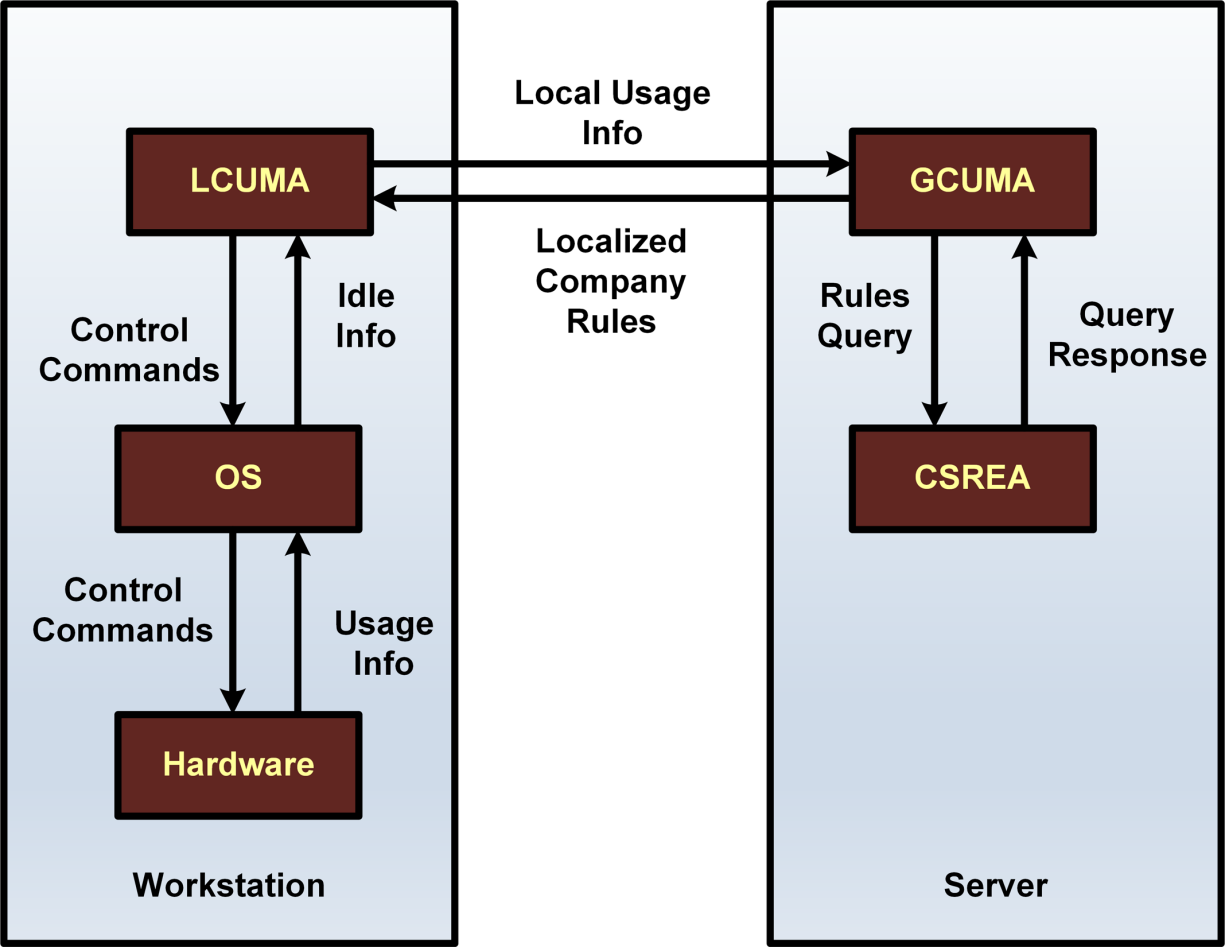
MASMINC Architeture.

The proposed MASMINC multiagent architecture is demonstrated in Fig 1. MASMINC has been designed specifically to autonomously monitor carbon footprint usage by turning idle computers off by using shutdown, sleep or hibernation modes intelligently according to company policy. While, an architecture is typically not associated with internal details, here we do present some possible implementation scenarios in the subsequent sections.

### Overview

Traditional green approaches focus on minimizing Carbon footprint by turning computers off but do not give ideas on how to do it in an automated manner. MASMINC allows for an ability to control computer usage on a corporate network using autonomous software agents while still allowing users to publish and enforce policies at the company level.

MASMINC is made up of heterogeneous agents that observe and act based on inputs from the network. A simple example could be if agents were to monitor the idle time processing of a system and compare it with any assigned threshold. Subsequently, specific agents can be asked to power off any given computer based on the corporate policy. It is important to also note here that the exact level of idle processing required by agents and similar minor implementation details are left for customization up to the company implementing MASMINC. E.g. Idle time processing can be either simply based on CPU usage or else it can be based on office timing such that during the office time, MASMINC agents would not shut computers down but during off hours, idle processing could be used to power them off. The intent of the specific approach taken in this paper is to present a generalized architecture rather than focusing only on a single implementation which would have required giving specific details pertinent only to a specific Operating System (OS) or a multiagent toolkit–thereby limiting practical implementations in the corporate world. This is important because in the corporate world, what works for one OS may certainly be unsuitable for another. This is also true for different versions of the same OS. Besides, there is no guarantee that a specific OS or a toolkit will be suitable for the needs of every company aiming to implement MASMINC in their environment.

While these software agents are intrinsically simple in design, it is commonly known that simple agents can interact to exhibit collective intelligence in the form of an artificial CAS which can exhibit emergent behavior at the global level. Emergence is a phenomenon also prevalent in nature where simple agents such as cells interact based on sensors and actuators using chemical flow passing through the cell walls. Still, these simple agents form massively complex dynamic systems i.e. complex multi-cellular life. Likewise, depending upon the implementation details and company requirements for MASMINC, the agents may be used to act as intelligently as required by choosing between different outcomes.

To summarize, goals of the agents can include (but are not restricted to) the follows:

1. Dissemination of company policy from network administrators to individual computers via servers on the network.
2. Enforce and propagate company policy to computers allowing for the emergent minimization of carbon footprint usage over the network.
3. Allowing for programmable approaches to minimize loss of user data based on company timings and policy while reducing the carbon footprint. Timed shutdowns can also be configured using MASMINC to minimize hardware damage or data loss. Furthermore, MASMINC agents can be programmed to use decision support to minimize false positives and also give automated recommendations for shutting computers down. The program can be designed to differentiate between various types and reasons for invoking a shutdown. Specified rules can govern invoking specific actions on a particular computer autonomously in various ways and any operation can be executed either at a specific time “t”, after a certain time duration “Δt” or else immediately.

### Modeling Exercise

In this subsection, we formally present the design of the MASMINC architecture. Here, we first define a MASMINC Scenario.

**Definition: MASMINC Scenario.** A MASMINC scenario *S*_*i*_ is formally defined as a vector of operation *O* and urgency *U* as follows:

$$S_{i}=<o,u>$$ (1)

Where

O={Shutdown,Restart,Standby,Hibernate},o∈O

$$U=\{t_{n},t_{f},t_{d}\},\;u\in U\;\text{with}$$

$$t_{n}=\text{Now}$$

$$t_{f}=\text{At a fixed time}$$

$$t_{d}=\text{After a duration}$$

A list of possible scenarios of MASMINC is summarized in tabular form in Table 1. Here, we can note that all four possible operations can be coupled with three types of urgencies to create a total of 12 possible cases:

**Table 1:** MASMINC Scenario.

Scenario	Operation	Urgency
1.	Shutdown	Right Now
2.	Shutdown	At a Specific Time
3.	Shutdown	After a Specific Time Duration
4.	Restart	Right Now
5.	Restart	At a Specific Time
6.	Restart	After a Specific Time Duration
7.	Standby	Right Now
8.	Standby	At a Specific Time
9.	Standby	After a Specific Time Duration
10.	Hibernate	Right Now
11.	Hibernate	At a Specific Time
12.	Hibernate	After a Specific Time Duration

#### Agent Design

As discussed earlier, while architecture does not focus on implementation details, our goal here is to assist the readers in implementing the concept of MASMINC. As such, here we also give some details of the agent-design. The exact number of these agents in a particular network is customizable. This allows for different implementation strategies and may vary based on each instance of the operational environment. However, in general, MASMINC agents are of the following three basic types and will be discussed in more details in the subsequent sections:

1. Global Carbon Usage Monitoring Agent (GCUMA),
2. Local Carbon Usage Monitoring Agent (LCUMA),
3. Company Specific Rules Enforcement Agent (CSREA).

Next, we discuss some of the key architectural details of MASMINC:

#### Customizability by Design

MASMINC has intrinsically been designed as a customizable architecture that may be implemented in any suitable corporate environment. All MASMINC rules can be placed in the form of a text file which can be maintained by the network administrator. The rule file is loaded by the CSREA. By decoupling the rules from the agent code in the form of a separate file, we have eliminated the requirement to re-compile agents if rules are to be changed. A new rule file may simply be placed in a particular folder under observation by the CSREA. CSREA will load updated rules and propagate them to other agents across the network. As such, in this paper, while we do not focus on a particular rule file or format, we do however give proposals for developing a suitable customized implementation of the rule file.

#### Autonomous Checking

MASMINC can be used to provide autonomous checking of system statistics such as idle time, number of processes, CPU usage, interactive input from user etc. The LCUMAs that reside on the machines can be implemented on a particular system to periodically check system statistics automatically in order to take appropriate decisions based on the company and user rules.

#### Central Logging of Energy Saving Data

It is proposed that a centralized “Energy Consumption” log be maintained at the server machine. This log can be a text file or it can be in the form of a database. The idea is to use GCUMA to perform centralized logging based on status updates from LCUMAs. The LCUMAs themselves can also be designed as stateless considering that there may not be any need to save localized information. If required, Computer usage history can also be saved in separate tables in addition to the current status of each system in the log.

#### Agent Communication

**Fig 2:**
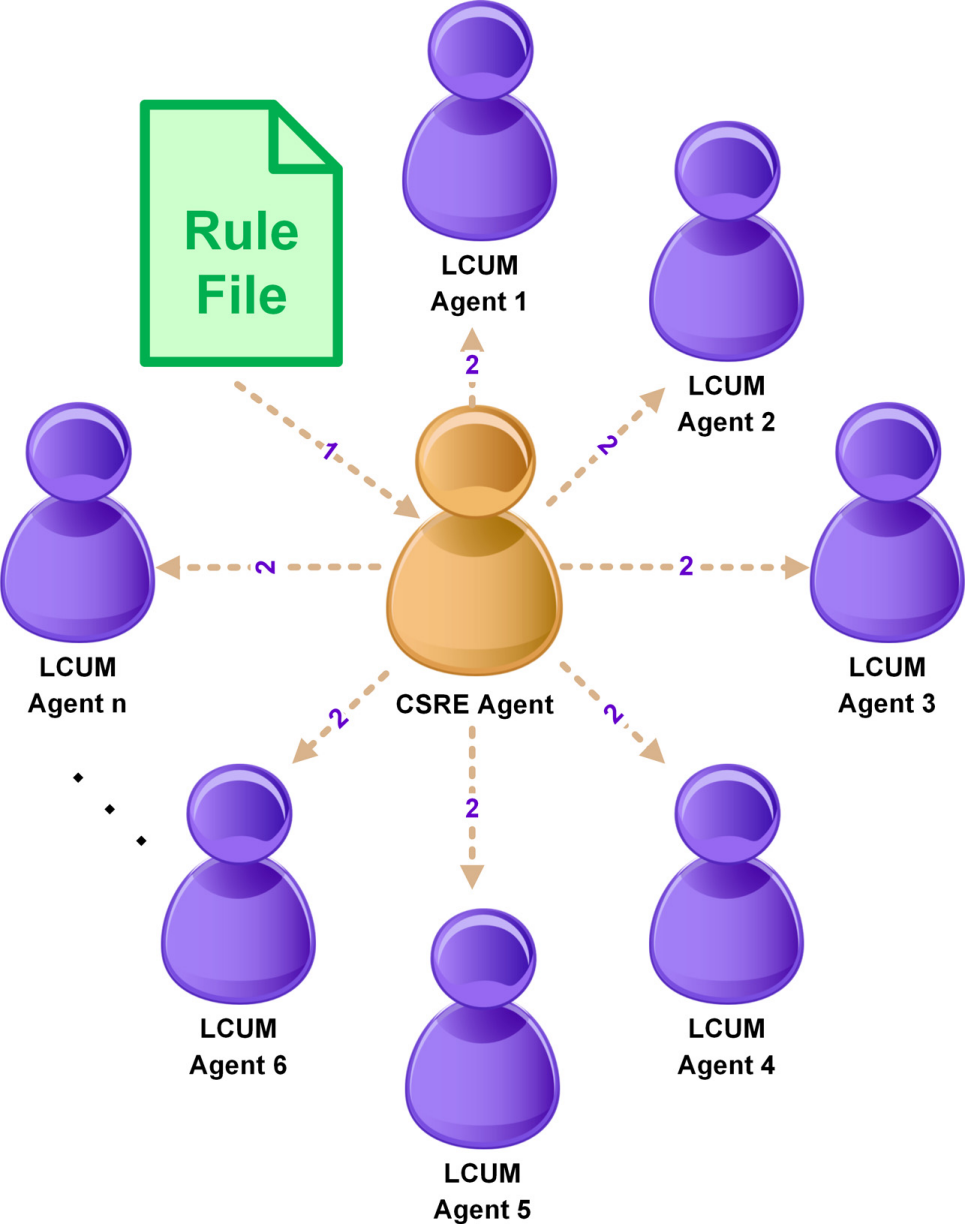
Communication between Agents of MASMINC and CSRE Agent.

**Fig 3:**
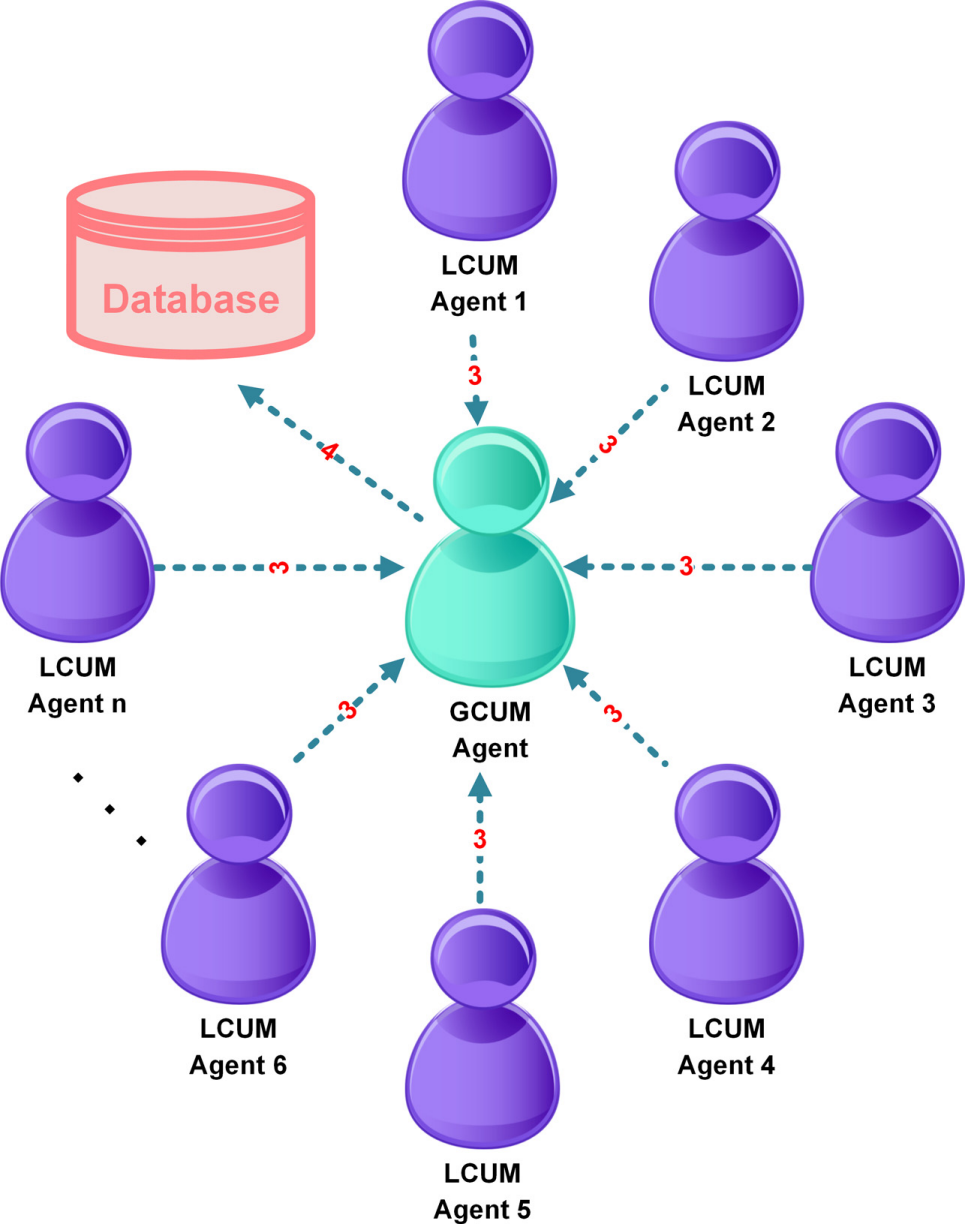
Communication between Agents of MASMINC and GCUM Agent.

Agents coordinate with each other using message passing. It is proposed that messages be designed to be of at least two key types: The first type is logging messages–messages which emanate whenever a computer is about to take some action. In addition, a second message type can be “control” messages–messages which can be used to advise agents to perform shutdown/standby or hibernation on the local machine. Fig 2 and Fig 3 represent the flow of messages between the agents of MASMINC. Table 2 shows the nature of messages that are exchanged between the agents of MASMINC.

**Table 2:** Types of Messages that are exchanged between MASMINC Agents.

Agent name	Send message to	Message Content
CSRE Agent	-	(1) Get rule file
CSRE Agent	LCUM Agent	(2) A rule file is sent
LCUM Agent	GCUM Agent	(3) Status update of corresponding machines is sent.
GCUM Agent	-	(4) Log information to the database

As can be noted here, GCUMA monitors the status of all other agents. Centralized logging allows the GCUMA to observe and 1maintain a complete status log of all computers. LCUMAs keep track of idle time and also periodically report machine status to GCUMA to ensure smooth network operation based on publicized rules. The CSREA loads administrative rules from the server machine and subsequently communicates these rules to various LCUMAs. As an example, rules may be designed such that a computer’s idle time may be used in conjunction with a threshold metric to make decisions in terms of the exact procedure to adopt for turning off the computer. While performing any local operation, a LCUMA also updates GCUMA about the status of the system. Thus, a GCUMA also serves as a bookkeeper for all machines managed by the MASMINC environment.

A detailed description of the working of MASMINC is as follows:

- Initially, the GCUMA and CSREA are created on the server machine (at boot up).
- Likewise, each machine managed by MASMINC creates LCUMAs at boot time.
- After boot, each LCUMA connects with the GCUMA and informs about the system.
- The CSREA extracts rules from the rule file. Subsequently these rules are sent to all LCUMAs.
- After the initialization of the system, i.e., the creation of GCUMA, CSREA and the LCUMAs, the LCUMAs start monitoring local resources by observing and keeping a short-term utilization record in the respective machines.
- The LCUMAs also periodically send status information back to the GCUMA regarding the energy usage, which includes system information such as system idle time, the system boot time, keyboard/mouse status etc.
- The GCUMA periodically updates this information to determine if the computer is idle or in use by a current active user in a database.

**Fig 4:**
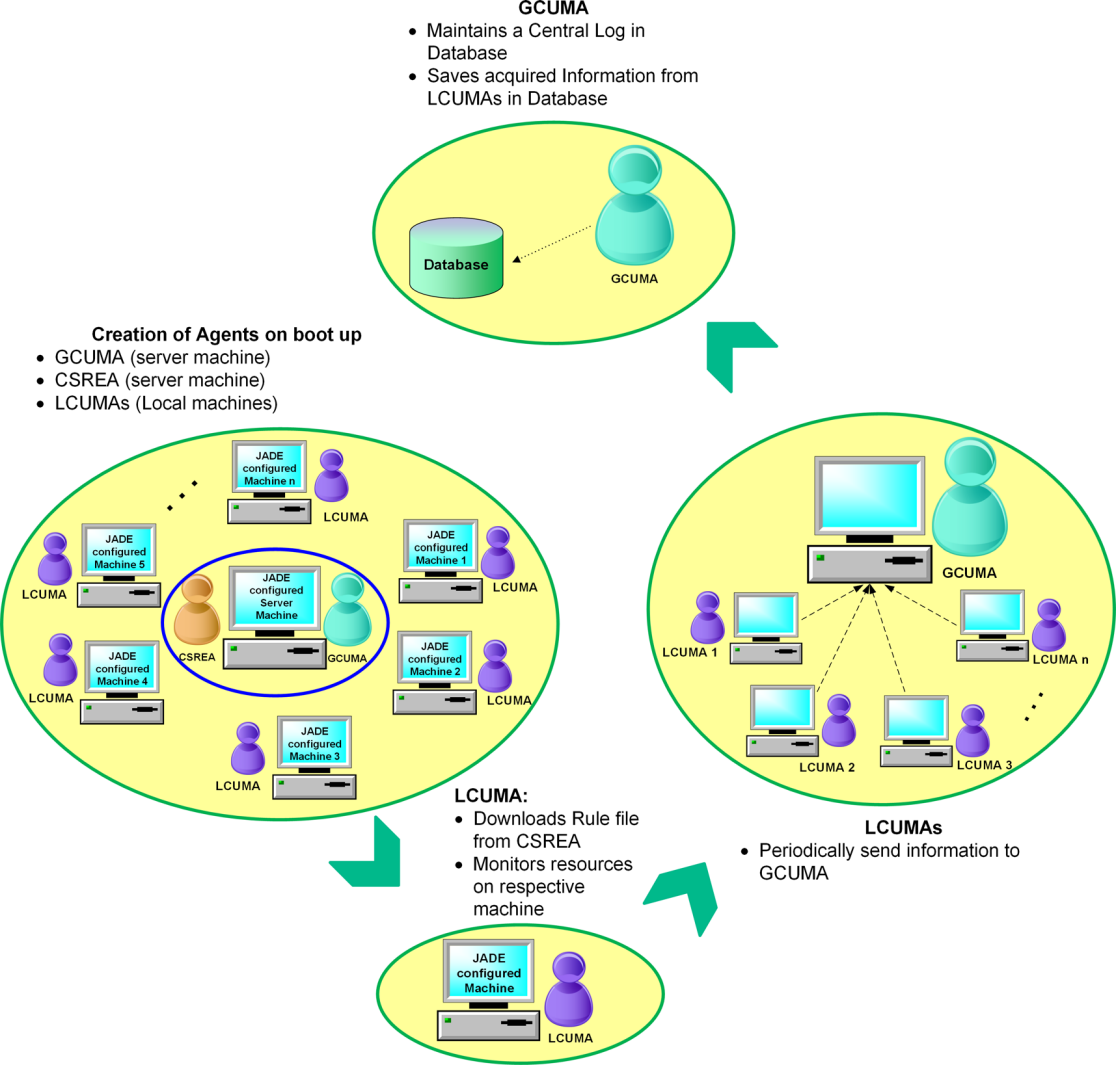
Illustration of the working of MASMINC (assuming JADE run-time).

Fig 4 depicts the detailed working of the system as described above. Here, we note that MASMINC can be implemented using any multiagent run-time. However, just to give an example, we present some ideas for using a popular toolkit such as Java Agent Development Framework (JADE) [41]. In this figure, we note that initially different agents are created at startup on various machines. Although client machines only execute LCUMAs, the server machine(s) execute GCUMA and CSREA.

#### Agent Environment

The concepts of the Agent Environment of MASMINC have been adapted from Russell and Norvig [42]. The dimensions along which task environments are categorized are given as follows:

- Fully Observable vs. Partially Observable
- Deterministic vs. Stochastic
- Episodic vs. Sequential
- Static vs. Dynamic
- Discrete vs. Continuous
- Single agent vs. Multiagent

The specific properties of MASMINC agents’ environment can thus be noted as follows:

1 Partially Observable

The environment itself is not fully observable. Besides, since other agents do not keep a state of their own; the GCUMA must keep an internal state to keep track of its environment.

2 Deterministic

The agent environment is deterministic, as the next state of the environment is completely determined by the current state and the action executed by the agent in a particular scenario is already specified in the rule file. The behavior of the agents is also predictable and is based on the inferences made using the rules file. The rules file provided by the CSREA agent can contain explicit sequence of actions that need to be accomplished in any particular course of events or they can instead actually contain rules which are usable for inference making using an appropriate expert system such as JESS [43].

Here, we would also clarify that the definition of “Deterministic” according to AIMA book by Russell and Norvig [42] entails that an environment is deterministic if the previous states completely determine the outcome of the next states. In other words, while the actions of the users may themselves be considered stochastic in nature, since the output state of the system is completely dictated by the previous state, hence we have labeled the task environment as deterministic.

3 Episodic

MASMINC agents have an episodic task environment as in each episode; the agent perceives the environment and then performs an appropriate action.

4 Static

The environment is static because the environment does not change its state except for the situation when any agent performs one of the expected actions.

5 Discrete

The environment is discrete in terms of the number of computers.

6 Multiagent

Since MASMINC involves sets of agents that cooperate to accomplish a common goal of managing carbon footprint, the environment is formally noted to be a multiagent environment as per the definition given in the above standard reference for intelligent agents.

#### Distributed Staggered CFPA Allocation Mechanisms

Now that we have talked about various agents, we now present strategies which may be used to allocate carbon footprint to individual computers. Once an amount of carbon footprint to a particular corporate environment has been allocated, five types of CFPA distribution schemes are proposed here as examples for usage in particular implementation–described as follows:

1. Constant Distribution of Carbon Footprint Allowance
2. Uniform Distribution of Carbon Footprint Allowance
3. Customized Distribution of Carbon Footprint Allowance
4. Triangular Distribution of Carbon Footprint Allowance
5. Hybrid Distribution of Carbon Footprint Allowance

**Fig 5:**
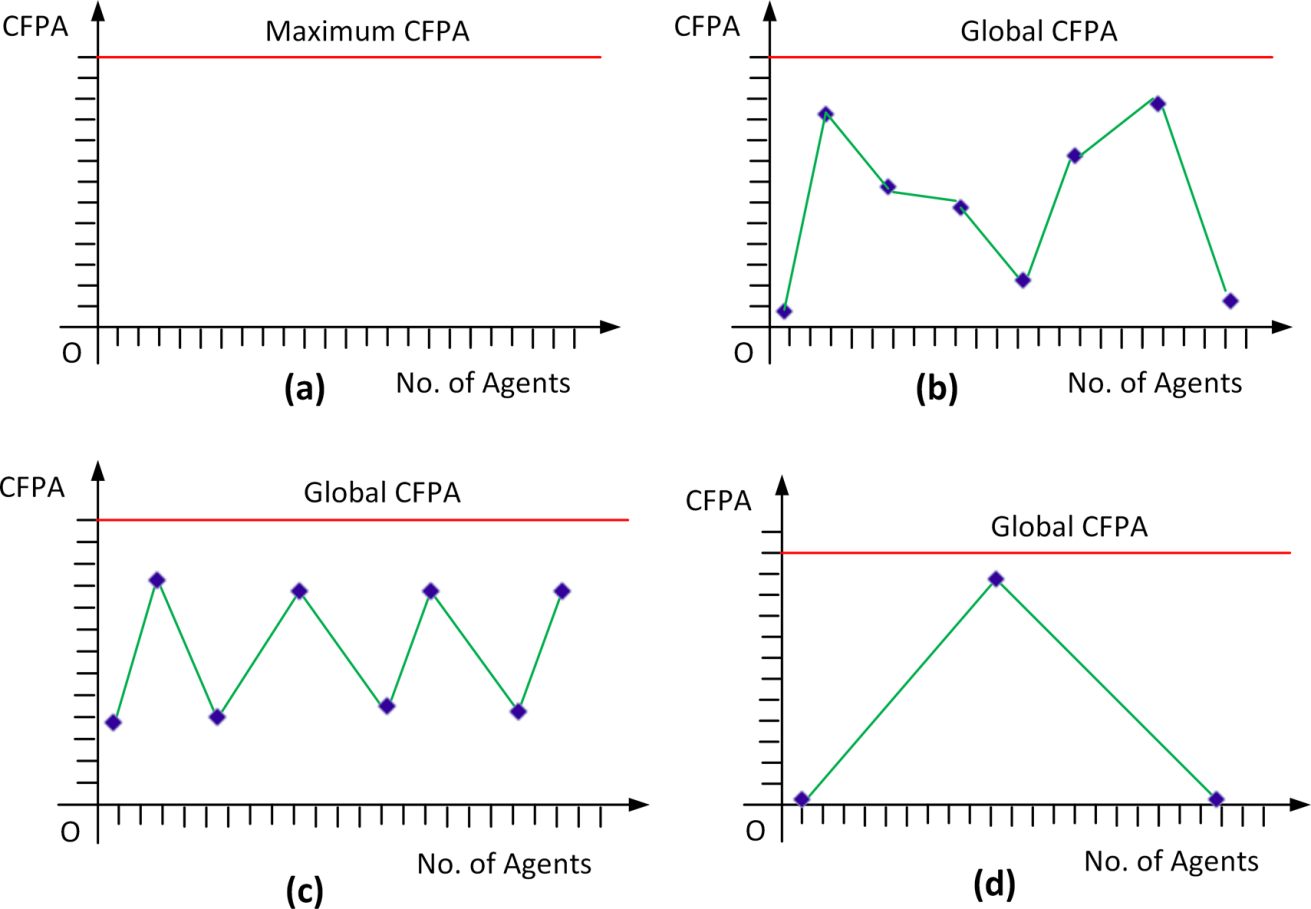
Different CFPA Distribution Schemes. (a) Constant Distribution of CFPA–All agents get the same amount of CFPA (b) Uniform Distribution of CFPA–Distribution of CFPA to agents is variable but all local values of CFPA should add up to the Global CFPA limit of the company (c) Customized Distribution of CFPA–Some random agents are assigned very higher values of CFPA (d) Triangular Distribution of CFPA–distribution of CFPA to agents follows Triangular distribution.

A graphical representation of four types of CFPA allocation schemes is given in. It should be noted here that the graphical representations of the CFPA allocation schemes are provided to Fig 5 give a general idea of the distribution schemes, thus figures are not drawn to scale.

The details of the above-mentioned distributions are given as follows:

1 Constant Distribution of Carbon Footprint Allowance

In this type of distribution, a constant amount of CFPA is apportioned to each computer. Fig 5(A) represents Constant Distribution of CFPA.

2 Uniform Distribution of Carbon Footprint Allowance

In uniform distribution of CFPA, a random amount of CFPA is allotted to each agent. However, there is a constraint on the distribution of random CFPA to the agents, i.e., the total random CFPA allocated to all the agents must be equal to the Global CFPA as specified by the company. Let LCFPA denote the Local Carbon Foot Print Allowance, GCFPA denotes the Global Carbon Foot Print Allowance with k denoting the total number of agents residing on the machines in a network then we can note that

$$GCFPA={\sum_{n=1}^{k}LCFPA}_{n}$$ (2)

This type of distribution is illustrated in Fig 5(B).

3 Customized Distribution of Carbon Foot Print Allowance

The Customized distribution is convenient in the sense that the desired amount of CFPA can be allocated to a specific machine. For example, a company might want to provide a relatively larger amount of CFPA to a server machine and smaller amounts of CFPA to the nodes. Thus, through this distribution, the CFPA allocation can be customized as desired by company policy. Customized Distribution is shown in Fig 5(C).

4 Triangular Distribution of Carbon Foot Print Allowance

In the Triangular distribution, there are three parameters that define the allocation: a maximum value, a minimum value and a mode value for CFPA allocation, giving rise to a triangular distribution. The mode value of CFPA is the most likely value. As an example, let’s say if we have a maximum CFPA value of 100, a minimum CFPA value of 10 and a mode CFPA value of 50, then this type of distribution will generate random samples with a minimum value of 10, most likely value of 50, and maximum value of 100 as shown in Fig 5(D).

5 Hybrid Distribution of Carbon Foot Print Allowance

As the name suggests, this type of distribution can be used based on a combination of other distributions. In particular, the possible usage of hybrid distribution of the CFPA could be based on a mixture of the triangular distribution and customized distribution.

#### Rule-based Administration

The idea of using rules developed from the actual MASMINC system can be realized by means of a text file (e.g., an XML file) that contains the rules for autonomous execution. The file is thus an imperative part of the system. Agents dynamically evaluate the state of machines using these rules specified in the file allowing them to take decisions in order to save carbon footprint usage without causing disruptions to working users.

There are clearly two distinct possibilities of hosting the administrative rule file. The first and more traditional approach to disseminating a rule file is to host it on each machine where it could be loaded by the agents at startup of the system. However, this approach has several inherent problems. Firstly, every time, the rule file changes, the administrator would have to manually upload the rule file to all machines. Secondly, there is a risk of manual editing by the user, which might end up inadvertently destroying important files by causing unnecessary shutdowns. Therefore, to minimize user errors as well as to provide a common interface for the autonomously execution of rules, we propose that the corporations implementing MASMINC may host the rule file on the server. After hosting, an agent can parse the rules from this file and the same may subsequently be propagated to other agents on the rest of the network computers.

Technically, this heterogeneous agent environment collectively forms a cooperative multiagent system [44]. In a cooperative multiagent environment, agents cooperate with each other towards a common goal. In the present system, this common goal is to autonomously limit the usage of Carbon footprint while minimizing any delays or data loss in personal computer usage on the network.

**Fig 6:**
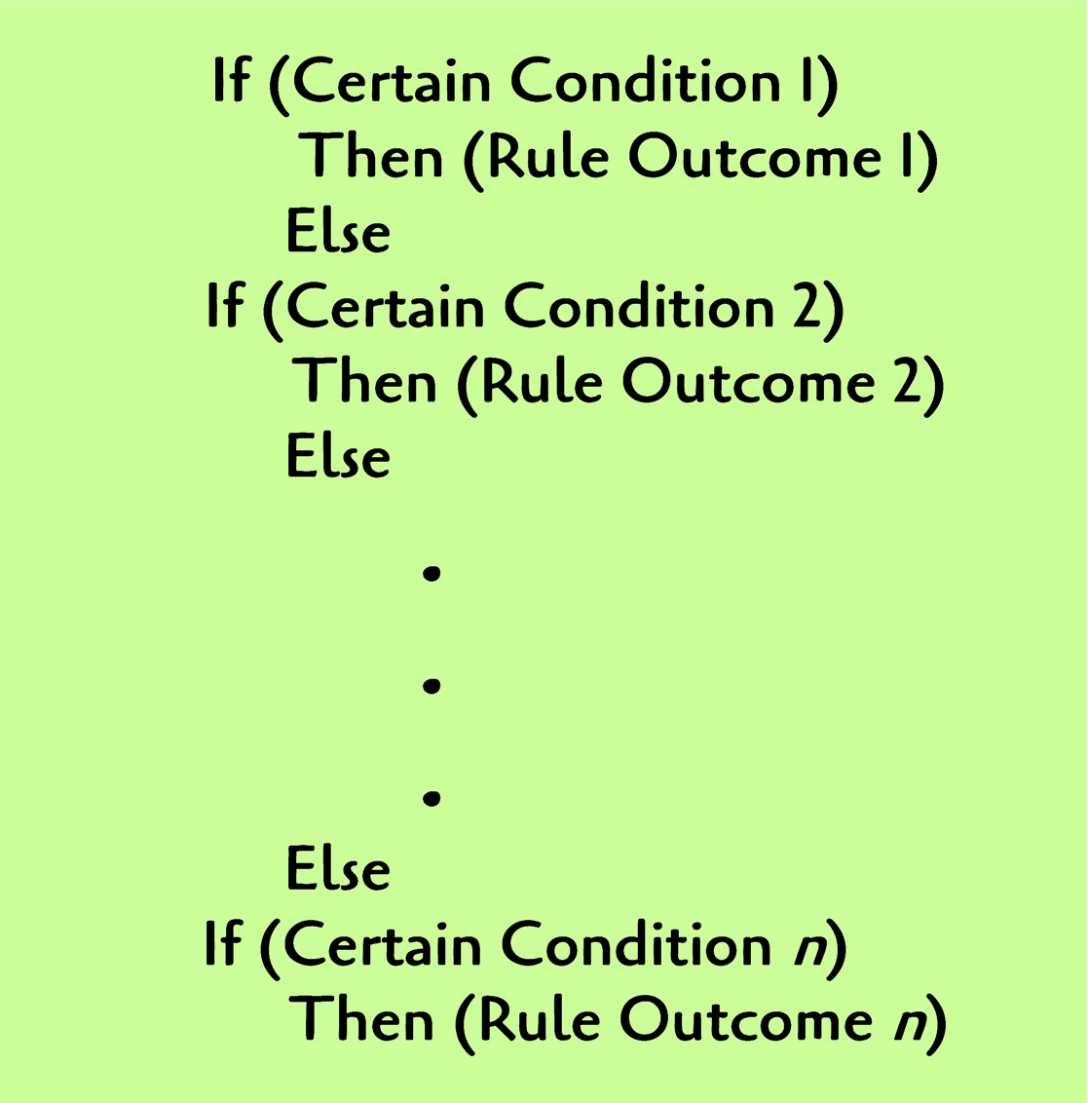
Examples of rule in a Rule File.

Fig 6 proposes example semantics of a rule file.

**Fig 7:**
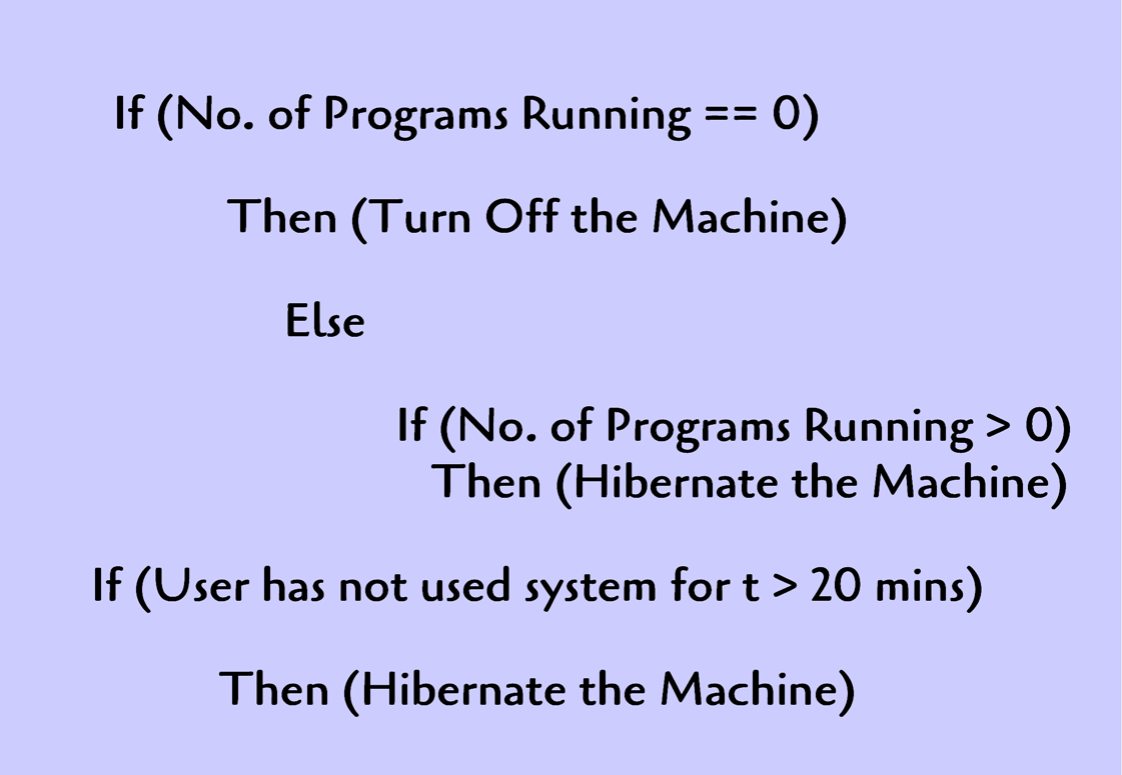
Example rules for turning off or hibernating a particular machine.

Fig 7 presents a set of examples of rules for turning off or hibernating machines.

### Experimental Setup for Validation

This section presents two experimental setups. In the first case, simulation experiments are presented for large-scale corporate networks using an ABM. Whereas subsequently, we give details on how an actual multiagent system may be practically implemented for the proposed MASMINC architecture. It is important to note here once again that our focus is on presenting an architecture which requires being platform and toolkit agnostic. Based on our previous experience in developing software multiagent systems, we believe that experienced software developers, should be able to use this detailed guidance to easily develop implementations of the MASMINC architecture, using any standard multiagent toolkit on a given version of an OS.

#### Agent-based Model Design

In this section we present an experimental setup for a large number of computers, i.e., up to 1000 computers, using the NetLogo Simulation Environment [45]. It is important to note here the importance of the simulation because it is impractical to actually perform experiments on corporate networks having more than 100 computers on physical hardware–thereby demonstrating the effectiveness of the agent-directed simulation approach.

6 NetLogo, An Introduction

NetLogo is a suitable candidate for multiagent simulation and has already been used in the multiagent literature for similar use. Choosing NetLogo to simulate MASMINC is backed by several reasons. Wilensky [46] notes that NetLogo is particularly well suited for modeling complex systems over time, making it a suitable choice as a simulator for MASMINC. In addition, considerable work has previously been published on the use of NetLogo and similar agent-based simulation toolkits for developing simulations of multiagent systems including[44, 47–49].

7 Implementation Details of Netlogo Model

The agents are created in NetLogo with the “breed” keyword. After defining the breeds, different breeds can be made to behave differently using NetLogo functions. The function “make-agents” is used to create and place all agents in the environment.

**Fig 8:**
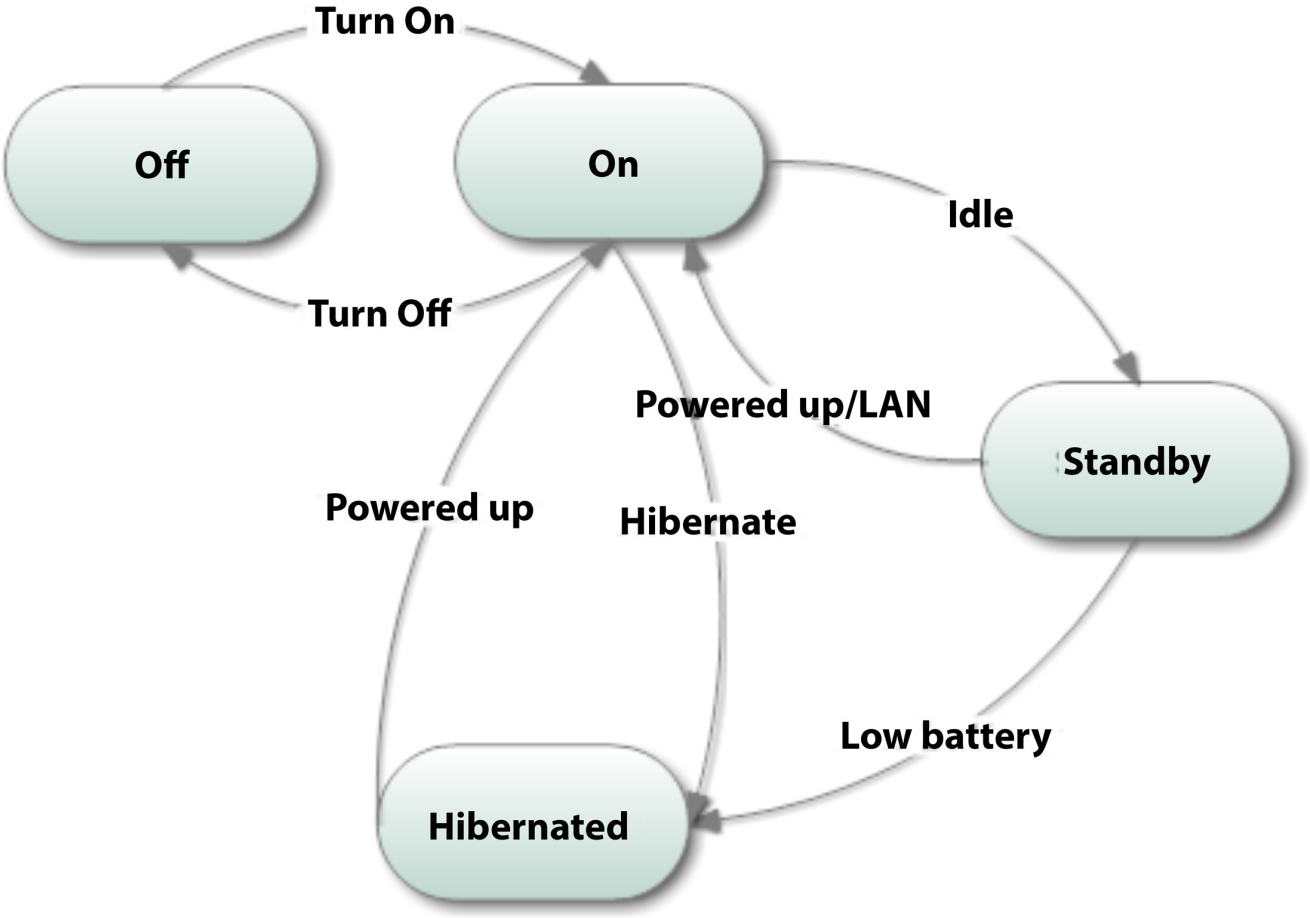
State transition model of a typical personal computer.

Here personal computers are modeled as agents and thus follow the state chart as noted in Fig 8. A machine can turn on and off. It can also go to the standby state if idle. Likewise, the computer can be hibernated or restarted.

#### Example of Using a Software MAS Toolbox

In this section, we present an example setup for MASMINC which multiagent researchers can easily develop for practical application in networks. While, as noted earlier, the basic design goal of MASMINC is to be platform and toolkit-agnostic, this setup is intended to be an example for researchers who develop their own practical solutions.

The agents in MASMINC may be implemented physically using a toolkit such as JADE. JADE is a software environment to build agent systems for the management of networked information resources. The JADE toolkit has been developed to model agents which comply with the Foundation for Intelligent Physical Agents (FIPA 2000) specifications for interoperable multi-agent systems [50].

- Messaging among the MASMINC Agents

Here we present example messages for MASMINC agents using JADE. As an example of sending messages from GCUMA to a particular LCUMA, example code is given as follows:

ACLMessage msg = new ACLMessage (ACLMessage.INFORM);

msg.setContent("Send the list of computer activity according to rule 1 of rule file");

AID LCUMA1 =…;

Msg.addReceiver(LCUMA1);

Send msg;

An example of receiving message from GCUMA to a particular LCUMA is given as:

ACLMessage msg = receive();

if (msg! = null)

<.... Report computer activities to the GCUMA…>

else

<… Monitor computer activities on the designated computer…>

Likewise, other important features of MASMINC can also be easily implemented in JADE or any other multiagent toolkit.

## Simulation Results

In this section, we present the simulation results using the agent-based model described earlier for validating MASMINC. A key goal of the simulation experiments was firstly to validate the benefits of MASMINC. Secondly, we wanted to evaluate if the benefits offered by MASMINC would hold with a variation in the number of managed computers. The simulation data is available online from the Dryad Digital Repository [51].

### Metrics

In this section, we describe the metrics followed by the experimental setup and a detailed discussion of the results. The key metric used for performance evaluation in this model is the Maximal Carbon Footprint Allowance (MCFPA). It is defined as *“The maximum carbon footprint of a computer if it were turned on for the entire day”–thereby representing the greenhouse gases for a single computer remaining in power-up state for a day*.

Having this metric is important because of several reasons:

1. In its absence, it is difficult to generalize how a single computer would behave.
2. There are no other known metrics of a maximum allowance for a single computer in a given corporate network.
3. Having a maximum allowance allows us to ensure that we evaluate and compare the performance of any appropriate scheme with the maximum. In other words, we can measure how much of the allowance is left over after each day for each scheme.

The maximum carbon footprint allowance (MCFPA) and its relationship with the daily carbon footprint allowance (DCFPA) can thus be expressed formally as given in the following set of equations:

$$CFPA_{\max}=24\times CFPA_{Single}$$ (3)

Here CFPA_max_ is the MCFPA, *N* is the total number of computers in the network and CFPA_Single_ is the allowance for one computer for 1 hour.

Assuming a unit value for CFPA_Single_ and using 3, we can express the “Daily Carbon Footprint Allowance” (DCFPA) as follows:

$$DCFPA=CFPA_{\max}-\sum_{t=0}^{t=23}n_{t}$$ (4)

It follows from 4 that

$$DCFPA=24\times N-\sum_{t=0}^{t=23}n_{t}$$ (5)

Where

*n*_*t*_ represents the average number of computers which remained on in the hour *t*.

Next, we can calculate the hourly change in Carbon Footprint Allowance as follows:

$$\Delta Allowance_{h}={\left(Allowance\right)}_{h-1}-{\left(Allowance\right)}_{h}$$ (6)

$$Hourly\;Change\;in\;Allowance=\Delta Allowance_{h}$$ (7)

Next, we present description of different experiments for the validation of the proposed MASMINC architecture.

### Modeling a typical Office Network

**Fig 9:**
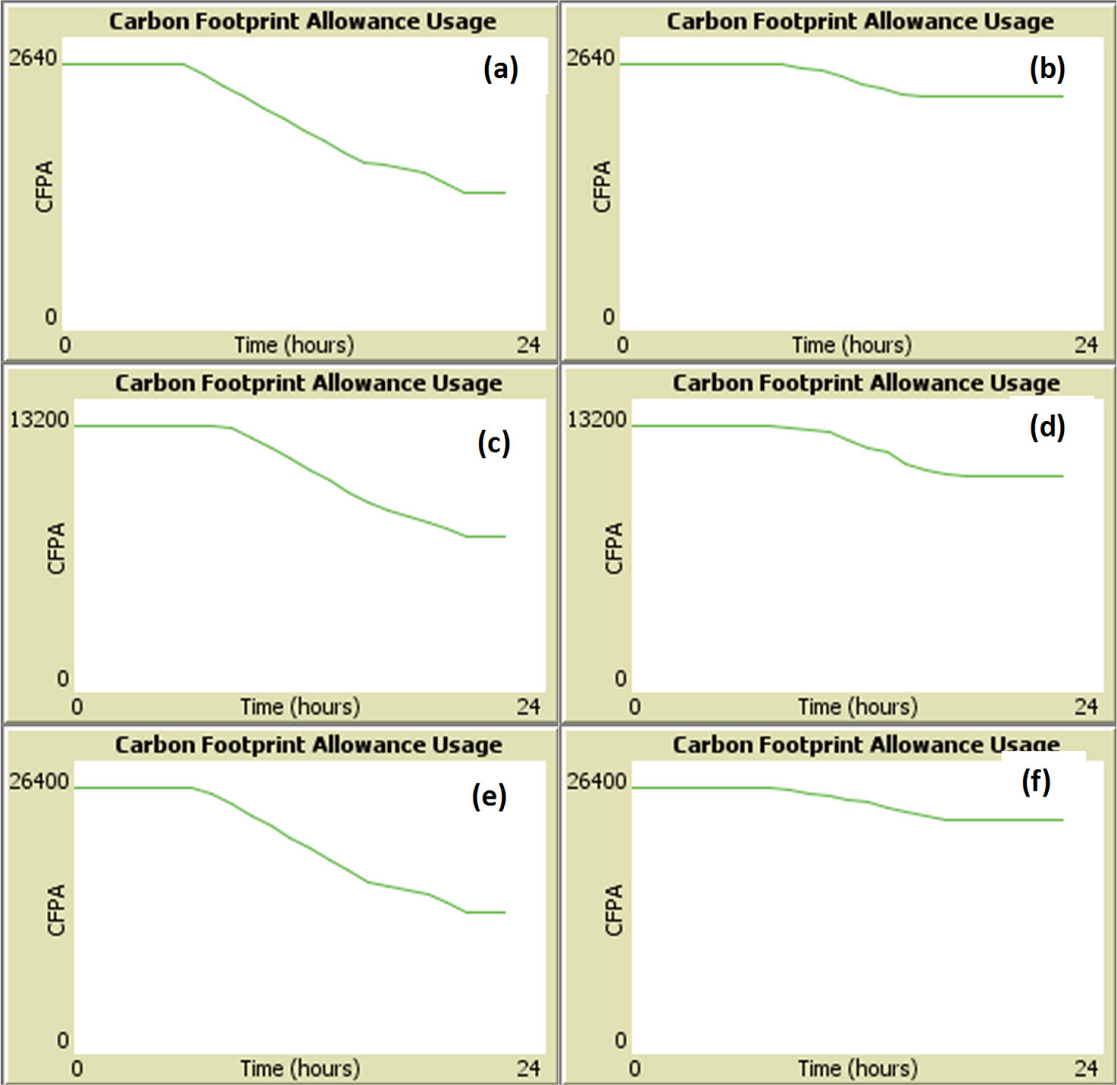
NetLogo Simulation plot showing the normal workplace carbon footprint Allowance usage in 24 hours of a day. (a) with n = 100 without MASMINC (b) with n = 100 with MASMINC (c) with n = 500 without MASMINC (d) with n = 500 with MASMINC (e) with n = 1000 without MASMINC and (f) with n = 1000 with MASMINC.

The first set of simulation experiments was to simulate the CFPA consumption in relation to the usage of computers in a typical corporate working environment. This experiment demonstrates how the simulation screen varies over a simulated typical day as can be seen in Fig 9. In the figure, the X-axis represents time in hours and the Y-axis represents the Carbon Foot Print Allowance. The numbers 0 and 24 on the x-axis represent 24 hours of a day whereas the numbers 2640, 13200, 26400 on the Y-axis is the maximum Carbon Foot Print Allowance that is associated with a specific company. Here we can notice that at the start of the day, computers are almost all turned off so leftover allowance is constant, i.e., the usage of CFPA amount is nearly negligible. As time passes, gradually some computers are on and some are turned off during office hours, resulting in lowering of the allowance, i.e., the usage of the allowed CFPA amount. During peak hours, almost all of the computers are in use, resulting in usage of a significant amount of the CFPA allowance. During breaks such as lunchtime, few computers may be in active use. This data has been simulated based on a typical office environment in the corporate world. However, it should be noted here that this particular setup has only been used as an example–other office cultures can also be easily modeled in the same simulation model.

### Variation of Carbon Footprint Allowance Usage with Number of computers

**Fig 10:**
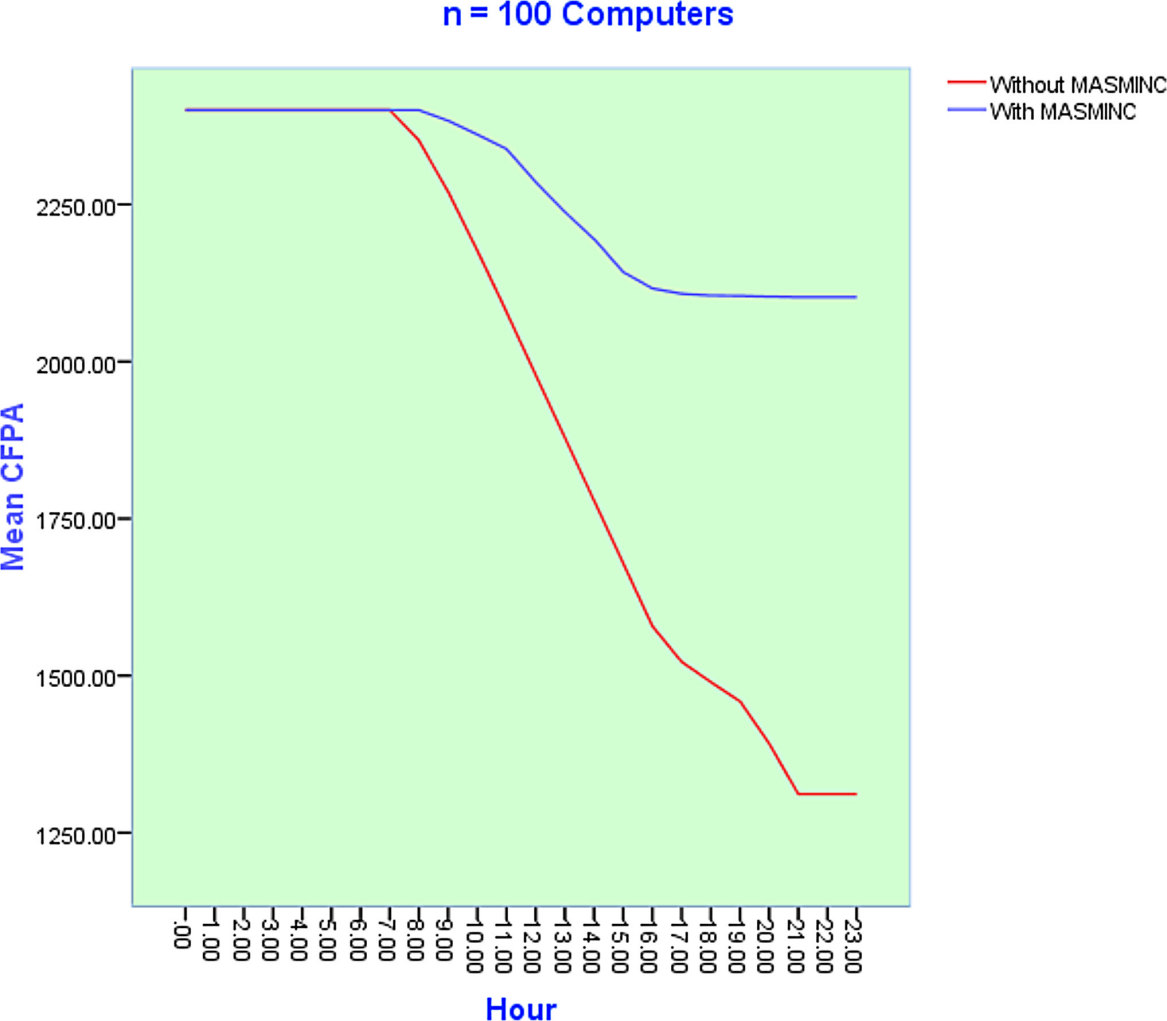
Results of 50 Simulation experiments with n = 100 computers during a day before and after applying the proposed MASMINC Approach.

**Fig 11:**
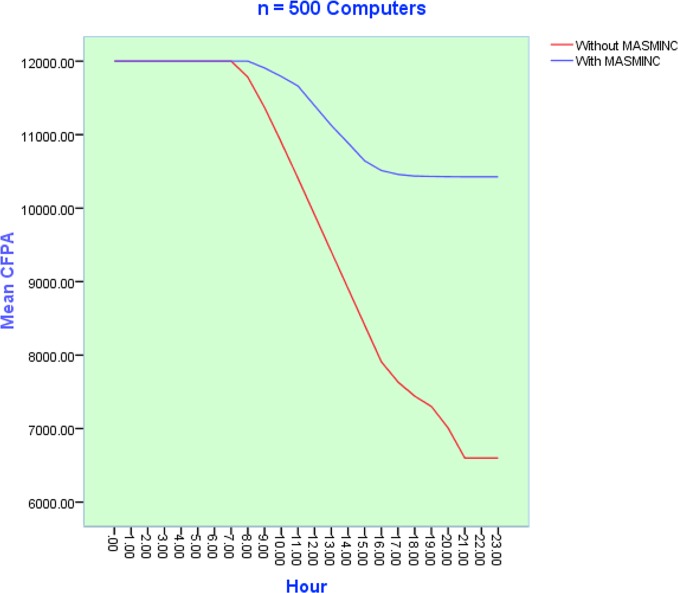
Results of 50 Simulation experiments with n = 500 computers during a day before and after applying the proposed MASMINC Approach.

**Fig 12:**
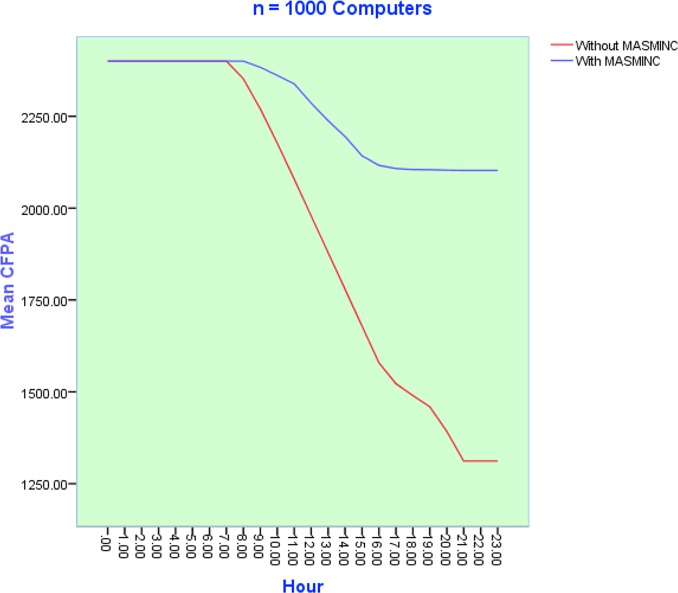
Results of 50 Simulation experiments with n = 1000 computers during a day before and after applying the proposed MASMINC Approach.

The objective of the second set of experiments was to discover how CFPA consumption varied with the number of computers in a company. To minimize the stochastic effects of simulation, each simulation experiment was repeated *50* times. Subsequently, results obtained from the simulation have been plotted on the plot. It is pertinent to note here that parameter sweeping has also been used. Fig 10, Fig 11 and Fig 12 illustrate the results of simulation experiments by varying the number of computers in the system. On the x-axis, we see the 24 hours while the y-axis shows the CFPA consumed by the computers. Initially, the maximum CFPA remains constant. This can be seen by the appearance of the initial straight line. The usage of computers increases with the passage of time. This usage causes a gradual decrease in the CFPA. The CFPA approaches zero, representing a scenario when a computer has used up the allowed CFPA. In other words, this situation now calls for action to be taken based on heuristics. This action could be a shut down or hibernation either immediately or after some time. The details of this shutdown are dictated by the company rules as can be publicized via the rule file. As can be seen in the three scenarios, the emergent patterns are almost the same for the number of computers ranging from N = 100, N = 500 and N = 1000. In all cases, MASMINC outperforms computers without MASMINC and a significant amount of leftover allowance is also available in all cases.

### Comparative Evaluation of MASMINC with Energy Star approach with change in the number of computers

The Energy Star program was introduced by the Environmental Protection Agency (EPA) of the United States for the promotion and identification of energy efficient products. The computer manufacturers focused on high performance keeping in view the energy efficiency constraint [52].

**Fig 13:**
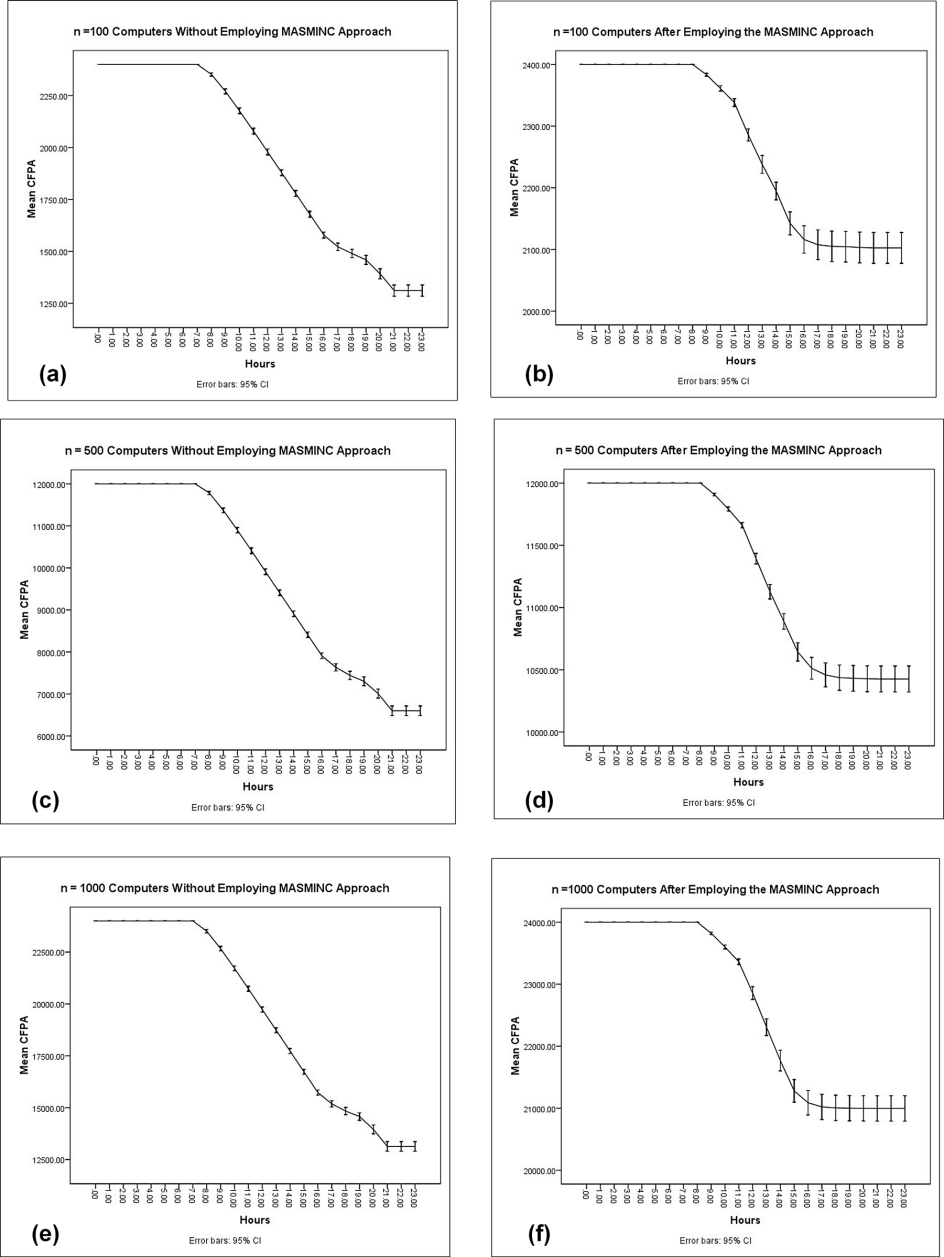
Results of 50 simulation experiments of daily Carbon Footprint Allowance Usage of computers ranging from 100 to 1000 during a day showing error bars with 95% Confidence Interval with Energy Star (Left) and with MASMINC (right).

In this section, we compare MASMINC with the Energy Star Model. Energy Star Model saves the energy of the system by simply shutting down the computers that are sitting idle without the consideration of the loss of user data. On the other hand, the MASMINC approach decides rationally which computers should be turned off. Here, we compare the effects of the Energy-Star approach to the MASMINC approach by means of varying the number of computers. We perform extensive simulation experiments by varying the number of computers from 100 to 1000. Each simulation was repeated 50 times and results were plotted using a 95% confidence interval as can be seen in Fig 13. Here it is pertinent to mention that if we examine the plots, we note a higher variance in the case of MASMINC. The variance is high because computers are being shutdown as per need. In the case of regular usage, the computers are kept turned on resulting in low variance. This however is actually besides the point; the important point to be noted here is actually that the Carbon footprint allocated in the case of MASMINC is saved more than without MASMINC even in extreme cases.

On the left side, the allowance is plotted with the use of Energy Star and on the right side, it is plotted using MASMINC. As can be seen, the number of computers that remain consuming power is significantly lower in the case of MASMINC as compared with the Energy Star approach.

### Discussion on why the MASMINC approach performs better than Energy*

It is clearly apparent that a blanket policy of shutting down computers is not a viable approach in a corporate environment. Now, let us examine the Energy Star approach which is mostly used for home computers and does not cater for corporate networks as seen in the simulation experiments in the previous section:

1. Firstly, Energy Star is an approach designed primarily for residential computers. By default, it does not offer a mechanism for a medium to large-sized company to enforce and implement a complex computer usage policy. Simple policies such as Energy star are thus not effective in this domain [53]. Complex situations can include employees being given short and hard deadlines. In these situations, the company would probably be more interested in accepting the extra usage of computers rather than forcing users to shut computers down to save the environment. Besides, in practice, this policy would anyway be impractical because system administrators cannot easily enforce such type of policies on large networks.
2. Secondly, suppose for a moment, even if there was a way of updating the Energy Star settings in a distributed manner, the user can simply change the times of shutting down or standby of the computer, thereby nullifying the effects of the company policy.
3. Thirdly, it is common experience that hibernation and standby procedures of personal computers are error-prone and can cause data loss or even cause significant disruption of work.

Thus, we can note here that while computers may be manually configured for Energy star to follow good energy usage practices–the approach is impractical from the standpoint of the corporate world and the larger a company network, the more visible this problem would be.

### Why an Agent-based Approach?

Here, in this section, we would like to elaborate exactly why an agent-based approach is more appropriate for the presented solution. The primary reason for using agents instead of simple software is that in any typical large corporation, rules governing the use of Computing resources can actually be extremely complex. If these rules were to be implemented in an architecture then of course such software agents can physically be implemented as software services. However, if we examine the design of such software, technically these are rational agents as per the definitions of Russell and Norvig in their renowned book on Artificial Intelligence [42]. Therefore, these rational agents can be designed for a particular corporation to use complex rules such as “only hibernate a computer if the user is not a Manager” or “turn off if the time is after regular work hours and the user has not used the computer for 20+ minutes and the company mode is currently “regular” in contrast to “Crunchtime” mode in which case the computer should not be hibernated” etc. It is following of these complex rules which requires these pieces of software to be termed specifically as agents as is norm in the research of this area.

## Conclusions and Future Work

In this paper, we have presented, we believe for the first time, the demonstration of Cognitive Agent-based Computing to model a Multiagent system for managing and minimizing Carbon-footprint in corporate networks. We have demonstrated how Exploratory Agent-based Modeling can help in modeling complex systems and scenarios in Communication Network architectures. MASMINC has been designed to autonomously monitor and limit Carbon footprint usage without causing a significant degradation of the user experience. Autonomous agents running on different computers perform various system maintenance tasks using rule files loaded on the server machine. We present the Exploratory Agent-based model demonstrating various simulation experiments using realistic data for a large variation of computers. Extensive simulation experiments demonstrated the effectiveness and scalable nature of the MASMINC approach in a variety of scenarios. The proposed MASMINC architecture is particularly useful for implementation in large corporate offices.

A limitation of our current work is that we have not evaluated the effects of MASMINC on the quality of service to the end users–being out of scope of this work. As such, a future direction could be to study an implementation of MASMINC in a particular environment to subsequently evaluate it in terms of how to better design MASMINC rules causing minimal disruption to end-users users or a loss of grade of quality of service experience. Another possible extension of the work could be to evaluate the use of MASMINC on the “Internet of Things” and “cloud computing” since visions of these areas envisage possibly extensive usage of data centers. Additionally, we believe that the use of autonomous architectures such as MASMINC on cloud computing is imperative for two key reasons. Firstly, the movement away from traditional desktop environments to mobile devices and servers to the cloud makes it an important case study. Secondly, the use of virtualization in cloud computing results in diverse energy patterns on physical servers. Prevailing trends also require servers to move to using low-power architectures like ARM that also have better (lower carbon emission) cooling requirements. Finally, more work needs to be performed to evaluate the effects of Carbon footprint allocation inside data centers to minimize “hot spots” due to localized allocation of work units to physically-close CPUs in data centers.
